# Infection by Third Persons

**Published:** 1897-12-04

**Authors:** 


### Infection by Third Persons.

An ingenious little suggestion is made by a medical officer of health who thinks that Section 126 of the Public Health Act, 1875, requires extending, so far as concerns penalties on exposure, so as to cover the cases of infection carried about by "third persons," such as, among others, "casual visitors to scarlet fever cases carrying away the seeds of the disease for communication to fresh victims."

Our first objection to any such proposal is that the law as it stands is sufficient to deal with any such cases as are at all likely to arise. Our next is that the carriage of infection by third persons, although a possibility in some cases, is an entirely minor and exceptional mode of distribution, and that, for this reason also, special legislation to prevent it is unnecessary; and our third is that any legislation of the sort would be applicable to many besides casual visitors, and if enforced, as all laws should be, would interfere with the ordinary business of life in a manner which would be quite unjustifiable unless the carriage of infection by third persons were proved to be a far more common event than we believe it to be.

If such proposals are to be carried into effect uniformly and without favour we may well pause to ask what they will lead to? As a carrier of infection it is quite clear that the casual visitor is harmless in comparison with, says the mother who has been nursing the sick child all day. Yet she goes out to do her marketing, or chats with the butcher boy at the door, or passes the time of day with the baker as he leaves his loaves. What then are we to do with her? The district nurse and the medical man will also be under an embargo, and it is clear that any legislation to control the casual visitor must also interfere with every detail of daily life, and throw us back to the times of the great plague when tradesmen delivered their goods by aid of a string from an upper window, and when payment was received in money thrown into a bowl of water.

But is all this fuss about infection by third persons necessary? The teachings of experience almost uniformly say it is not. Of course, every one admits that infection can be carried by means of fomites, and that if a doctor sits upon his patient's bed, and rubs his head against him in listening to his lungs, or gets his hands soiled with infectious material while inspecting his throat, and then straightway goes and rubs himself against another patient, he may carry the disease. But suppose he does not do these things, and that he properly washes his hands—that is, the parts that have actually touched the patient—all experience goes to show that this infection by third persons is a mere bugbear.

It is perfectly well known that in general hospitals which receive infectious diseases, and in which, even if these are placed in separate wards, the doctors and the students pass direct from one ward to another, any diffusion of the disease throughout the house is of the rarest possible occurrence, while, we would ask, how often is it not the case that medical men take their own children with them on their rounds without any harm resulting? One of the most eminent of the physicians practising in London used to take his children with him in his carriage when he visited the fever hospital, and either leave them at the door or let them play in the garden till he came out, when—soaked with infection, as some people would say—he would get into the carriage and drive away with them. Did they then catch scarlet fever? Not at all. Yet they were susceptible, for later on, after running all these "risks" without any harm, they did in the end catch the disease, not, however, while visiting the fever hospital, but while in the country, where they had been sent one year for the usual change of air! Let us put on one side all this dread and fear of infection by third persons. Such a thing may happen now and then, but it is not thus that infection is ordinarily spread, unless, perhaps, we make an exception in favour of varicella and of small-pox in unvaccinated communities.

We speak thus plainly because medical men, and especially medical officers of health, have shown themselves all too ready to encourage what we believe to be totally unnecessary restrictions of the ordinary work of daily life. If infection by third persons is an important factor in the dissemination of disease, let us face the matter and ask Government to provide for a special set of doctors and of nurses to look after all such infectious cases, and to make proper arrangements for their being fed and taken care of so long as they remain so dangerous to the community. But if such a mode of spread is only of occasional occurrence, do not let us pass silly laws. We may perhaps best judge of the manner in which laws to regulate the movements of "casual visitors" to cases of scarlet fever would operate by recalling the very patent fact that the medical man is exactly such a casual visitor. Already people are to be found who are quite ready to attribute their illnesses to infection carried by the doctor, and we may imagine the endless actions at law which would be hurled at his devoted head if such prejudices were crystallised into form by legislative enactments.

